# CD34+CD146+ adipose‐derived stromal cells enhance engraftment of transplanted fat

**DOI:** 10.1002/sctm.19-0195

**Published:** 2020-06-15

**Authors:** Mimi R. Borrelli, Ronak A. Patel, Charles Blackshear, Stephanie Vistnes, Nestor M. Diaz Deleon, Sandeep Adem, Abra H. Shen, Jan Sokol, Arash Momeni, Dung Nguyen, Michael T. Longaker, Derrick C. Wan

**Affiliations:** ^1^ Hagey Laboratory for Pediatric Regenerative Medicine, Department of Surgery, Division of Plastic Surgery Stanford University School of Medicine Stanford California USA; ^2^ Stanford Institute for Stem Cell Biology and Regenerative Medicine Stanford University School of Medicine Stanford California USA

**Keywords:** adipose‐derived stromal cell, autologous fat grafting, lipoaspirate, proangiogenic, regeneration, regenerative, retention

## Abstract

Fat grafting is a surgical technique able to reconstruct and regenerate soft tissue. The adipose‐derived stromal cells (ASCs) within the stromal vascular fraction are believed to drive these beneficial effects. ASCs are increasingly recognized to be a heterogeneous group, comprised of multiple stem and progenitor subpopulations with distinct functions. We hypothesized the existence of an ASC subpopulation with enhanced angiogenic potential. Human ASCs that were CD34+CD146+, CD34+CD146−, or CD34+ unfractionated (UF) were isolated by flow cytometry for comparison of expression of proangiogenic factors and endothelial tube‐forming potential. Next, lipoaspirate was enriched with either CD34+CD146+, CD34+CD146−, CD34+ UF ASCs, or was not enriched, and grafted beneath the scalp skin of immunodeficient CD‐1 Nude mice (10 000 cells/200 μL/graft). Fat retention was monitored radiographically more than 8 weeks and fat grafts were harvested for histological assessment of quality and vascularization. The CD34+CD146+ subpopulation comprised ~30% of ASCs, and exhibited increased expression of vascular endothelial growth factor and angiopoietin‐1 compared to CD34+CD146− and CD34+ UF ASCs, and increased expression of fibroblast growth factor‐2 compared to CD34+CD146− ASCs. The CD34+CD146+ subpopulation exhibited enhanced induction of tube‐formation compared to CD34+CD146− ASCs. Upon transplantation, fat enriched CD34+CD146+ ASCs underwent less resorption and had improved histologic quality and vascularization. We have identified a subpopulation of CD34+ ASCs with enhanced angiogenic effects in vitro and in vivo, likely mediated by increased expression of potent proangiogenic factors. These findings suggest that enriching lipoaspirate with CD34+CD146+ ASCs may enhance fat graft vascularization and retention in the clinical setting.

## Graphical abstract

CD146+ adipose‐derived stromal cells express increased levels of proangiogenic factors VEGF, ANGPT1, FGF and enhance tube‐formation by vascular endothelial cells. Fat grafts enriched this subpopulation also undergo the least resorption and possess improved histologic quality and vascularization. Enriching lipoaspirate with CD146+ ASCs may enhance fat graft retention in the clinical setting.

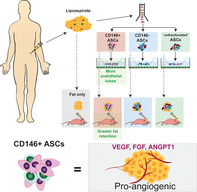

**Significance statement**

This study has identified a subpopulation of adipose‐derived stromal cells (ASCs), positive for the surface marker CD146, that have increased expression of proangiogenic genes and enhanced capacity to induce endothelial‐tube formation. Upon transplantation, fat enriched CD146+ ASCs underwent less resorption and had improved histologic quality and vascularization. The authors believe they have identified a subpopulation of ASCs with enhanced angiogenic effects in vitro and in vivo, and that enriching lipoaspirate with CD146+ ASCs may enhance fat graft vascularization and retention in the clinical setting.

## 1 INTRODUCTION

Autologous fat grafting (AFG) is an increasingly popular reconstructive technique; more than 75 000 AFG procedures were performed in 2018 in the United States alone.^1^ Initially popularized for its ability to restore soft tissue volume and soften contour deformities, fat has become increasingly appreciated for its regenerative potential. Today AFG is also used to improve the quality of scarred skin, facilitate wound closure, and reverse the effects of radiation‐induced soft tissue damage.^2^, ^3^ The adipose‐derived stromal cells (ASCs) within the stromal vascular fraction (SVF) of fat are thought to drive these regenerative effects.^4^ Evidence in support for this hypothesis has stemmed from several clinical and preclinical studies that demonstrate, compared to fat alone, grafts enriched with ASCs, also known as cell‐assisted lipotransfer, undergo less resorption and have improved vascularization and histological structure.^5^, ^6^, ^7^

Recent work has indicated that ASCs are a heterogeneous population comprised of distinct subpopulations of cells with differing functional capacities; bone morphogenetic protein receptor (BMPR)‐1A identifies ASCs with proadipogenic qualities^8^ and low expression of CD105 (endoglin) at the cell surface identifies ASCs with enhanced osteogenic capacity.^9^ Further defining ASC heterogeneity may improve the efficiency of current fat grafting procedures. Given that grafted fat often undergoes significant resorption, with retention rates ranging from 25% to 80%,^10^ and studies suggesting a lack of blood supply as a contributing factor, enriching grafted fat with ASCs exhibiting proangiogenic effects may promote early revascularization and thus better retention. Interestingly, a recent report identified CD146+ mesenchymal cells isolated from human umbilical cords that possessed enhanced ability to promote blood vessel maturation.^11^ We therefore hypothesized a similar existence of a subpopulation of ASCs with enhanced angiogenic potential that can increase the viability of fat grafts.

## 2 MATERIALS AND METHODS

### 2.1 Human adipose‐derived SVF harvest

Fresh human lipoaspirate was obtained from five healthy female donors with no medical comorbidities (age range: 18‐62 years) under the Stanford Institutional Review Board approval (IRB: 2188). The fat was washed with phosphate‐buffered saline (PBS, Thermo Fisher Scientific, Waltham, Massachusetts, Cat#10010023) and allowed to settle for 30 minutes at 4°C to separate into layers of lipid, fat, and blood. The layer of fat was retrieved with a serological pipette for both isolation of ASCs and for fat grafting. For the fat grafting experiment, a single source of fat was used; 4 mL of fat was set aside for grafting and the remaining fat was digested. The strategy to isolate CD34+CD146+ ASCs was adapted from methodology previously described.^12^, ^13^, ^14^ The fat was firstly enzymatically digested using type II collagenase digestion buffer (3000 U/mL, Sigma‐Aldrich, St. Louis, Missouri, Cat#C6885) supplemented with 100 U/mL DNase I (Worthington, Columbus, Ohio, Cat#NC9199796) in Medium 199 (Sigma‐Aldrich, Cat#M3769) at 37°C under gentle agitation for 30 minutes. The enzyme was then neutralized with fluorescence‐activated cell sorting (FACS) buffer (1X PBS with 2% heat‐inactivated fetal bovine serum [FBS, Thermo Fisher Scientific, Cat#A3840001], 1% Polaxamer 188 [P188, Sigma‐Aldrich, Cat#P5556], and 1% Penicillin‐streptomycin [PS, Gibco, Waltham, Massachusetts, Cat#5140122]), in a 1:1 ratio. The solution was then filtered through a 100 μm pore cell strainer (Corning, New York, Cat#431752), and centrifuged (5 minutes, 4°C, 450 rcf). The supernatant was aspirated, and the cell pellet was resuspended in 500 μL FACS buffer and carefully added as a layer to the top of 1 mL of Histopaque‐1077 (1.077 g/mL, Sigma‐Aldrich, Cat#10771) and centrifuged (1500 rpm, 15 minutes, room temperature [RT], no deceleration) for density separation. The cell layer (buffy coat) was carefully retrieved by pipette, washed by dilution in FACS Buffer, and recentrifuged (450 rcf, 5 minutes, 4°C) to derive the SVF pellet.

### 2.2 FACS isolation of subpopulations of ASCs

**FIGURE 1:**
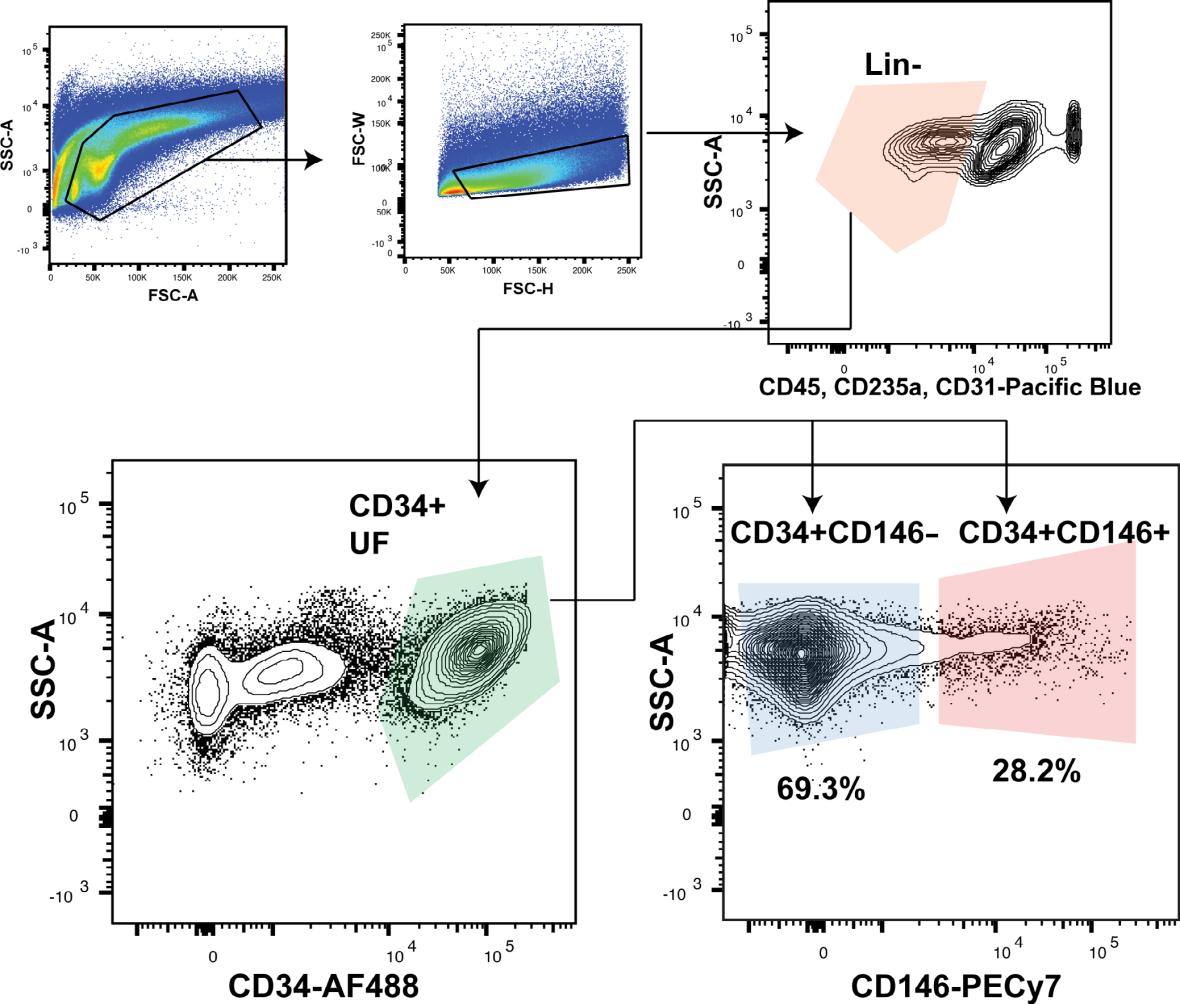
Gating strategy used to isolate CD34+CD146+, CD34+CD146− and CD34+ UF ASCs. A Lin‐negative (CD45−CD235a−CD31−) and positive gating strategy was used to isolate three populations of ASCs: CD34+CD146+, CD34+CD146−, and CD34+ UF ASCs. CD34+CD146+ comprised 28.2% and the CD34+CD146− 69.3% of CD34+ ASCs, respectively. ASC, adipose‐derived stromal cell; UF, unfractionated

Freshly harvested stromal vascular cell (SVC) human lipoaspirate cell pellets were resuspended in FACS buffer and stained with antibodies specific to human: CD45‐Pacific blue (PB, BioLegend, San Diego, California, Cat#304022) for hematopoietic cells; CD235a(Glycophorin A)‐eFluor 450 (BioLegend, Cat#349108) for erythrocytes, and erythroid progenitors; CD31(PECAM‐1 or platelet endothelial cell adhesion molecule)‐eFluor 450 (BioLegend, Cat#303114) for endothelial cells; CD34‐AlexaFluor(AF)‐488 (BioLegend, Cat#343517) for ASCs; and CD146‐PE/Cy7 (BioLegend, Cat#361007) for the proangiogenic ASC subset. All primary antibodies were stained in a 1:100 ratio, which was determined based on prior experiments. 4′,6‐diamidino‐2‐phenylindole (DAPI, Thermo Fisher Scientific, Cat#D1306) was used as a live‐dead stain. Flow cytometry (FACS Aria II, BD Biosciences, San Jose, California) was used to isolate three populations of ASCs based on a Lin‐negative (CD45−CD235a−CD31−) and positive gating strategy; CD34+CD146+, CD34+CD146−, and CD34+ unfractionated (UF) ASCs (Figures 1 and S1).^15^, ^16^, ^17^ Cells were sorted using a 100 μm nozzle, and a flow rate of 1, with the “purity” setting. Purity was tested before sorting by analyzing 2000 freshly sorted cells and was found to be >99% for each of the three ASC subpopulations.

### 2.3 Angiogenic gene expression

To confirm proangiogenic gene expression, ASC subpopulations were freshly sorted directly into TRIzol (Invitrogen, Carlsbad, California, Cat#15596026). RNA was extracted using the RNeasy Mini Kit (Quiagen; Hilden, Germany, Cat#74104). Reverse transcription was performed using Reverse Transcription Reagents (Invitrogen). Quantitative real‐time polymerase chain reaction (qRT‐PCR) was performed using an ABI Prism 7900HT Sequence Detection System (Applied Biosystems, Foster City, California) with Power SYBR Green PCR Master Mix (Applied Biosystems, Cat#4367659) as the reporter. Gene expression levels of three proangiogenic genes (vascular endothelial growth factor [VEGF], angiopoietin‐1 [ANGPT1], fibroblast growth factor‐2 [FGF]) were assessed by qRT‐PCR. Expression levels of all genes were normalized to glyceraldehyde 3‐phosphate dehydrogenase expression values for statistical analysis. Significant differences in gene expression levels were determined using the relative threshold cycle method.^18^ To obtain ΔCT values, averaged cycle threshold (CT) values of the reference transcripts were subtracted from cycle threshold (CT) values of the candidate transcripts.

### 2.4 Angiogenic protein expression

Enzyme‐linked immunosorbent assays (ELISAs) for human vascular endothelial growth factor A (VEGFA) (Abcam, Cambridge, United Kingdom, Cat#119566), ANGPT1 (Abcam, Cat#ab99972), and FGF (Abcam, Cat#ab99972) were performed on the supernatant of FACS‐sorted CD34+CD146+, CD34+CD146−, and CD34+ UF ASC subpopulations that had been cultured (1000 cells/24‐well) in ASC media (10% FBS, 1% antibiotic‐antimycotic [Gibco, Cat#15240062], 1% GlutMax in Dulbecco's modified eagle medium [ThermoFisher, Cat#10569010]), at low oxygen conditions (2% O_2_ and 7.5% CO_2_) for 3 days. Standard protocols were followed, as per the manufacturer's instructions.

### 2.5 In vitro endothelial tube formation assay

**FIGURE 2:**
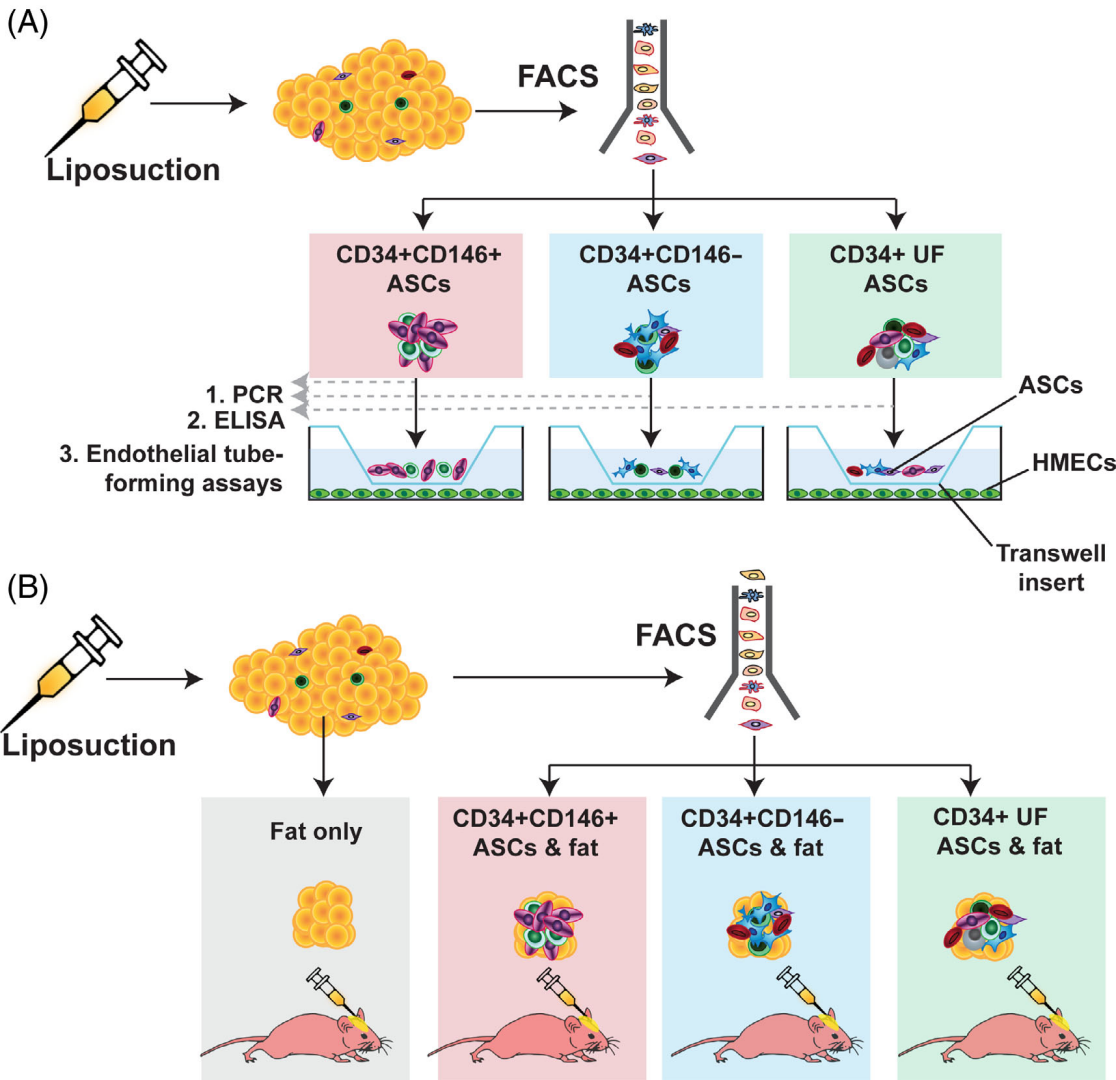
Overall experimental approach. CD34+CD146+, CD34+CD146−, and CD34+ UF ASCs were isolated by FACS for: A, gene expression analysis by PCR (1), protein expression analysis by enzyme‐linked immunosorbent assays (2), and endothelial tube forming assay in transwells with HMECs (3); and for, B, exploration of proangiogenic qualities of three ASC subpopulations in vivo by combining with fresh lipoaspirate and grafting into CD1 Nude mice (10 000 cells/200 μL lipoaspirate/graft). A group of mice receiving lipoaspirate with no ASCs (fat only) (200 μL/graft) served as a control. ASC, adipose‐derived stromal cell; FACS, fluorescence‐activated cell sorting; HMEC, human microvascular endothelial cell; PCR, polymerase chain reaction; UF, unfractionated

To explore the proangiogenic behavior of the ASC populations in vitro, endothelial tube forming assays were performed (Figure 2A). Human microvascular endothelial cells (HMECs) were purchased (ThermoFisher, Cat#C01125PA) and seeded (1.2 × 10^5^ cells/6‐well) on Geltrex (ThermoFisher, Cat#A1413201)‐coated 24‐well plates and cultured in endothelial cell media^19^ (Media 199 [M199] with 20% FBS, 2 mM glutamine [Thermo Fisher Scientific, Cat#25030081], 1% PS). FACS‐isolated CD34+CD146+, CD34+CD146−, and CD34+ UF ASCs were plated in ASC media and expanded to passage 1 at low oxygen conditions (2% O_2_ and 7.5% CO_2_). The ASCs were then directly seeded in cell culture inserts (7.5 × 10^3^ cells/insert). All groups were cultured in triplicate. Three separate wells of endothelial cells were treated with 10 ng/mL recombinant VEGF (ThermoFisher, Cat#PHC9391) alone as a positive control. Endothelial‐ACS cultures were incubated for 18 hours and then the endothelial cells were stained with Calcein AM (Thermo Fisher Scientific, Cat#C3100MP) immediately prior to imaging. Imaging was performed using a Leica DM5000 B Light microscope (Leica Microsystems, Buffalo Grove, Illinois) and a ×10 objective. Tubule analysis was performed using ImageJ software (https://imagej.nih.gov/ij/) and the Angiogenesis Analyzer tool which measures the total area of tube formation/mesh area, master segments which consist of pieces of the network delimited by two junctions, and master junctions which are intersections linking at least three master segments in each field of view.

### 2.6 Animals

Adult female 60‐day‐old CD‐1 Nude immunocompromised mice (Crl:CD1‐Foxn1nu, Charles River) were used for experimentation (total n = 32). Mice were maintained at the Stanford University Research Animal Facility (5 animals/cage) in sterile microinsulators and were given water and rodent chow ad libitum, in accordance with Stanford University guidelines. All experiments were performed in accordance with the Stanford University Animal Care and Use Committee Guidelines under an approved APLAC protocol (APLAC‐31212).

### 2.7 Fat grafting

To explore the proangiogenic effect of the ASC populations in vivo, fat grafting was performed. The three ASC subpopulations (CD34+CD146+, CD34+CD146−, CD34+ UF) were FACS‐isolated as described above. Four groups of mice were grafted with 200 μL of undigested human lipoaspirate either: (a) enriched with CD34+CD146+; (b) enriched with CD34+CD146−; (c) enriched with CD34+ UF ASCs; or (d) not‐enriched (fat only) (n = 8 per group, 10 000 cells/200 μL lipoaspirate graft) (Figure 2B). Fat was grafted directly into the subcutaneous space of the calvarium using methodology previously described.^7^, ^20^, ^21^ In brief, the fat grafts were first transferred to a 100 mL syringe. The mice were then anesthetized (2.5% isoflurane), placed in the prone position, and the calvarial region was sterilized using alcohol and betadine wipes. A 5 mm horizontal incision was made in the skin overlying the skull between the ears with straight scissors. A subcutaneous tunnel was created using blunted forceps, and the 200 μL fat grafts were injected in a retrograde fashion into the space. The skin was closed with a single horizontal mattress suture (6‐0 Polyamide 6, Ethilon black 18' P‐1 cutting, Johnson & Johnson, New Brunswick, New Jersey, Cat#697H).

### 2.8 CT scans and reconstructions

Micro‐computed tomography (microCT) imaging was performed using a Bruker Skyscan 1276 microCT (Bruker), as previously described.^22^ Immediately following fat grafting, mice were imaged to determine baseline fat graft volume. Thereafter, serial imaging was performed every 2 weeks for a total of 8 weeks postgrafting. Three‐dimensional reconstructions were performed using cubic‐spline interpolation to determine fat graft volume as a percentage of the original transplanted volume.^5^ All reconstructions were performed blinded by two investigators (M. R. B. and S. V.) and the mean of each score was calculated.

### 2.9 Fat graft harvest and histological analysis

After 8 weeks postgrafting, the mice were euthanized, and fat grafts were explanted and immediately fixed in 4% paraformaldehyde for 16 hours at 4°C. Samples were then washed with PBS, dehydrated in gradients of alcohols, and embedded in paraffin blocks. Blocks were sectioned into 8 μm slices and stained with H&E (Cat#H‐3502, Vector Laboratories, Burlingame, California) to assess for histological structure and inflammation, and Masson's Trichrome (Abcam, Cat#ab150686) to assess for fibrosis. Stained slides were imaged in bright field using a Leica DM5000 B Light microscope (Leica Microsystems; Buffalo Grove, Illinois) and the ×10 and ×20 objectives. Ten random sections were chosen per mouse per group for histological analysis and scoring. Four blinded, independent investigators (M. R. B., S. V., R. A. P., N. M. D. D.) evaluated fat graft quality according to a previously published scoring system which grades: (a) integrity (presence of intact, nucleated fat cells); (b) cyst/vacuoles; (c) inflammation; and (d) fibrosis.^23^ Scores range from 0 to 5; 0 indicates “absence,” and 5 indicates “extensive presence.” The mean scores per group across all investigators were calculated for statistical comparison. For immunostaining, samples were blocked (1X Power Block Universal Solution, BioGenex, Fremont, California, Cat#HK083) for 1 hour at RT, and then incubated with anti‐CD31(platelet endothelial cell adhesion molecule‐1 or PECAM‐1, Abcam, Cat#ab28364, 1:100) primary antibody to assess fat graft vascularization, anti‐perilipin‐1 (Abcam, Cat#ab3526, 1:100) to assess adipocyte survival/death, and anti‐F4/80 (Abcam, Cat#ab100790 1:100) to label macrophages, for 16 hours at 4°C. Slides were then washed with PBS and incubated with Alexa Fluor conjugated secondary antibodies (Donkey Anti‐Goat IgG Alexa Fluor 647, Abcam Cat#ab150135, 1:500; Donkey Anti‐Rabbit IgG Alexa Fluor 488, Abcam Cat#ab150073, 1:500; Goat Anti‐Mouse IgG Alexa Fluor 594, Abcam Cat#ab150120, 1:500) for 1 hour at RT. Slides underwent a final wash with PBS and were mounted in Fluoromount‐G with DAPI (Thermo Fisher Scientific, Cat#00‐4959‐52) to visualize cell nuclei. Fluorescent images were captured with laser scanning confocal microscopy using a Leica TCS SP8 confocal microscope (Leica Microsystems, Wetzlar, Germany). A uniform frame size of 1024 × 1024 was used with the ×25 or ×63 oil objectives. Quantification of fat graft vascularization was performed on fat grafts from five mice per group. To ensure large areas of the fat graft were assessed, tiled (3 × 3) *z*‐stacked (8 μm deep) fluorescent images were taken. To quantify graft vascularization, the percent of pixels positive for CD31 staining was calculated for five representative regions of interest (ROIs) of equal area using ImageJ software (https://imagej.nih.gov/ij/) following methods previously described.^24^, ^25^, ^26^, ^27^, ^28^ A threshold was used to select pixels occupied by blood vessels, represented by CD31 staining, and the image was binarized by converting the blood vessels to white (pixel value 255) and background pixels to black (pixel value 0) to form a “mask” of positive CD31 staining. The mean number of pixels positive for CD31 per area was calculated, and the average of the five ROIs per image were calculated to give one value per image.

### 2.10 Statistical analysis

Continuous data were described using the mean and SD of the mean when parametric, and with the median and the range when nonparametric. Data were reported as frequencies when categorical. Analysis of variance and Bonferroni multiple comparisons were used to compare means between groups. A *P* value of <.05 was considered significant. All statistical analyses were performed using Prism GraphPad 5.0 (GraphPad Software, Inc., La Jolla, California) statistical software.

## 3 RESULTS

### 3.1 CD146 identifies a proangiogenic subset of human ASCs

**FIGURE 3:**
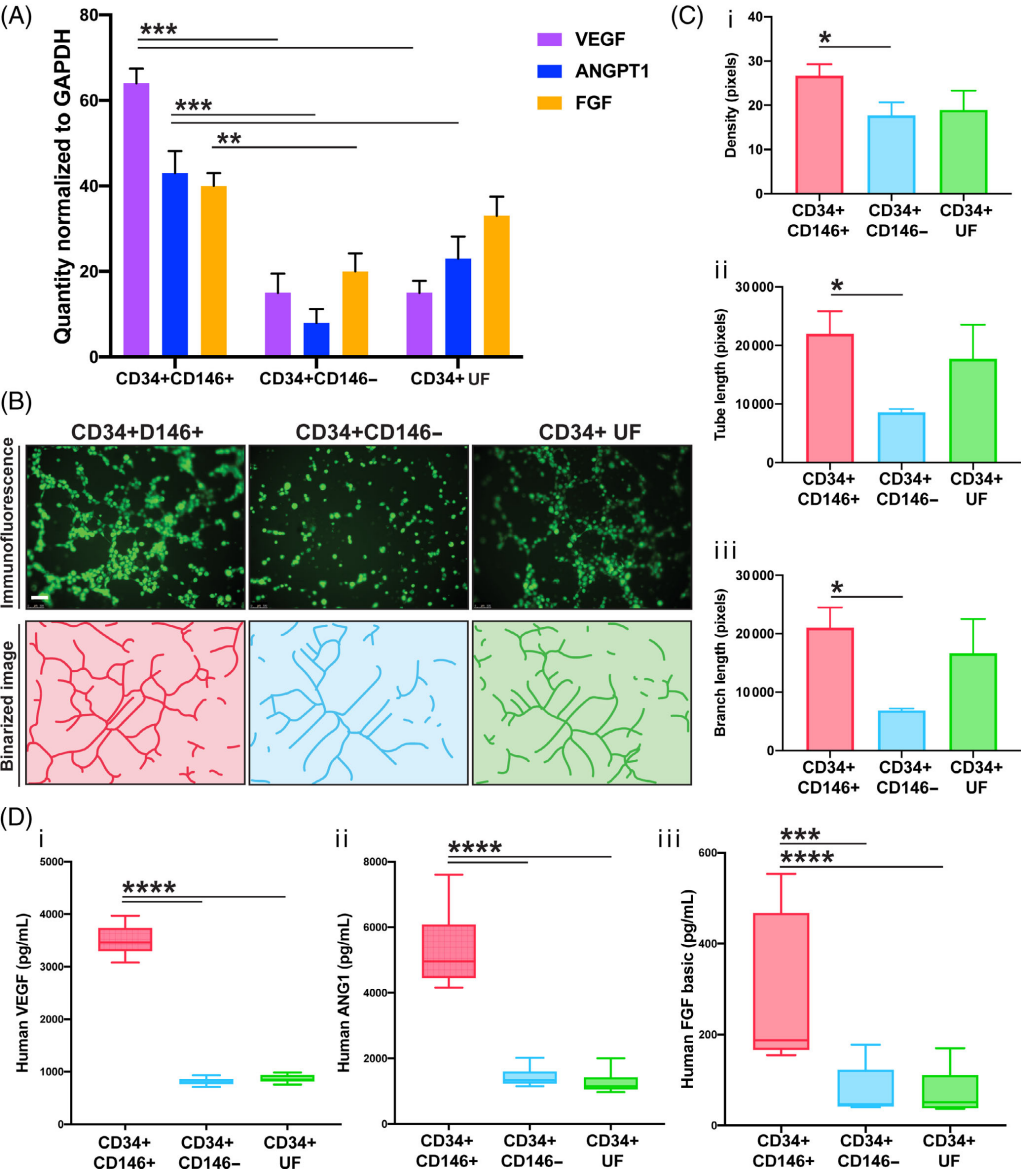
Gene expression, protein expression, and endothelial tube forming assays. A, Expression of three potent proangiogenic genes in CD34+CD146+, CD34+CD146−, and CD34+ UF ASCs was compared by PCR. CD34+CD146+ ASCs expressed significantly more VEGF (***P < .001) and ANGTP1 (***P < .001) than CD34+CD146− and CD34+ UF ASCs, and significantly greater levels of FGF than CD34+CD146− ASCs (**P < .01). B, Representative immunofluorescence images (top row) and binarized images from ImageJ analysis (bottom row) showing results from endothelial tube forming assays. Human microvascular endothelial cells (green due to staining with Calcein AM) were cocultured for 18 hours in transwells with CD34+CD46+ ASCs (left), CD34+CD146− ASCs (middle), and CD34+ UF ASCs (right). Scale bars = 100 μm. C, Graphs showing results from endothelial tube forming assay analysis demonstrating, C(i), greater total pixel density, C(ii), tube length, and, C(iii), total branching length in HMECs cocultured with CD34+CD146+ compared to CD34+CD146− ASCs (all *P ≤ .05). D, Graphs showing the results from enzyme‐linked immunosorbent assays comparing protein expression between three ASC subpopulations. CD34+CD146+ ASCs expressed significantly more VEGF (****P < .0001) (D[i]) and ANGTP1 (****P < .0001) (D[ii]) than both CD34+CD146− and CD34+ UF ASCs, as well as significantly greater levels of FGF than CD34+CD146− ASCs (***P < .001) and CD34+ UF ASCs (****P < .0001) (D[iii]). ANGPT1, angiopoietin‐1; ASC, adipose‐derived stromal cell; FGF, fibroblast growth factor‐2; HMEC, human microvascular endothelial cell; PCR, polymerase chain reaction; UF, unfractionated; VEFG, vascular endothelial growth factor

FACS analysis revealed that almost 30% of ASCs (CD34+ SVCs) were positive for CD146 (Figure 1). The digestion yielded ~380 000 CD34+ ASCs, and therefore ~114 000 CD34+CD146+ ASCs per 100 mL lipoaspirate. Gene expression analysis by PCR revealed CD34+CD146+ ASCs expressed significantly greater levels of VEGF (***P < .001) and ANGPT1 (***P < .001) than CD34+CD146− or CD34+ UF ASCs, and significantly greater levels of FGF than CD34+CD146− ASCs (**P < .01) (Figure 3A). After 18 hours of coculture, CD34+CD146+ ASCs induced HMECs to form significantly more tubes than did CD34+CD146− ASCs (Figure 3B), demonstrated by greater pixel density staining (Figure 3C[i]), greater tube length (Figure 3C[ii]), and more tube branching (Figure 3C[iii]) (all *P < .05). These results were also reflected at the protein level. The CD34+CD146+ subpopulation expressed significantly more VEGF (****P < .0001) and ANGPT1 (****P < .0001) than both CD34+CD146− and CD34+ UF ASCs, and significantly more FGF than CD34+CD146− (***P < .001) and CD34+ UF ASCs (****P < .0001). Recently, a mesenchymal stromal cell (MSC) derived from pericytes that is CD146+ but CD34− has been described and shown to have increased angiogenic potential,^12^, ^29^ enhances tissue repair, and promotes angiogenesis in vivo.^30^ We therefore compared gene expression between CD34+ ASCs (the classic adipose MSCs), CD34+CD146+ ASCs, and CD34−CD146+ pericytes (Figures S1 and S2A). Interestingly, gene expression was comparable between CD34+CD146+ ASCs and the CD34−CD146+ pericytes for VEGF and FGF, but the CD34+CD146+ ASCs had significantly higher expression of ANGPT1 (*P < .05) (Figure S2B).

### 3.2 Enriching fat grafts with CD34+CD146+ improves retention

**FIGURE 4:**
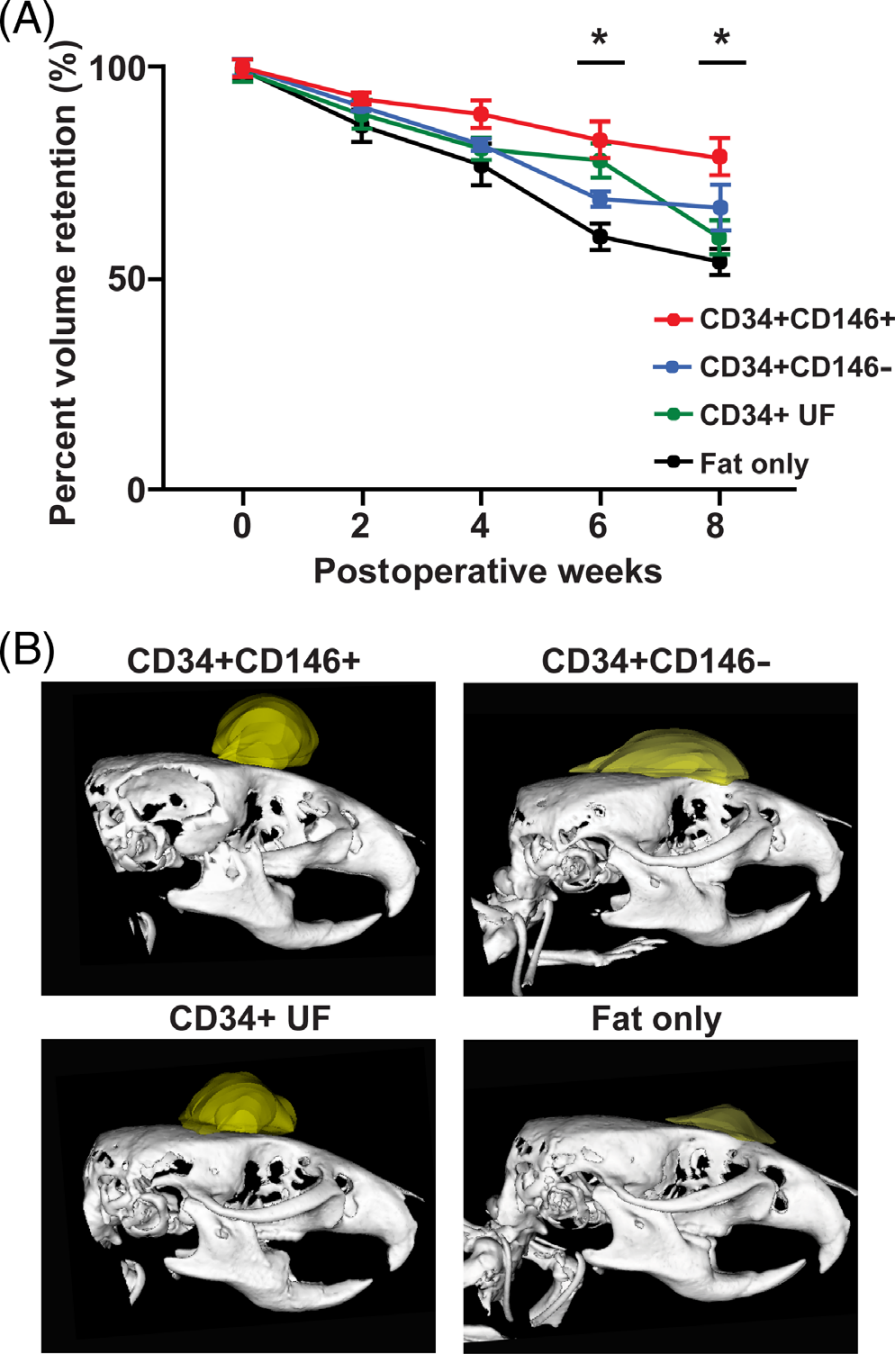
Fat graft retention. A, micro‐computed tomography, tracking across 8 weeks postgrafting revealed that fat grafts enriched with CD34+CD146+ ASCs underwent significantly less resorption than grafts enriched with CD34+CD146− or CD34+ UF ASCs, or fat transplanted alone. This trend reached significance at 6‐ and 8‐weeks postgrafting (*P < .05). B, Representative images showing volume of fat grafts of mice receiving lipoaspirate enriched with CD34+CD146+ (top left), CD34+CD146− (top right), and CD34+ UF (bottom left) ASCs, or fat only (bottom right) 8 weeks postgrafting, reconstructed from micro‐computed tomography data. ASC, adipose‐derived stromal cell; UF, unfractionated

Fat retention was monitored radiographically by Micro‐CT every 2 weeks for 8 weeks in total. Results indicated that fat grafts enriched with CD34+CD146+ ASCs underwent less resorption than grafts enriched with CD34+CD146− or CD34+ UF ASCs and fat alone. This trend became significant at 6 weeks (*P < .05) and remained significant 8‐weeks postgrafting (*P < .05) (Figure 4).

### 3.3 Improved histological quality of fat grafts enriched with CD146+ ASCs

**FIGURE 5:**
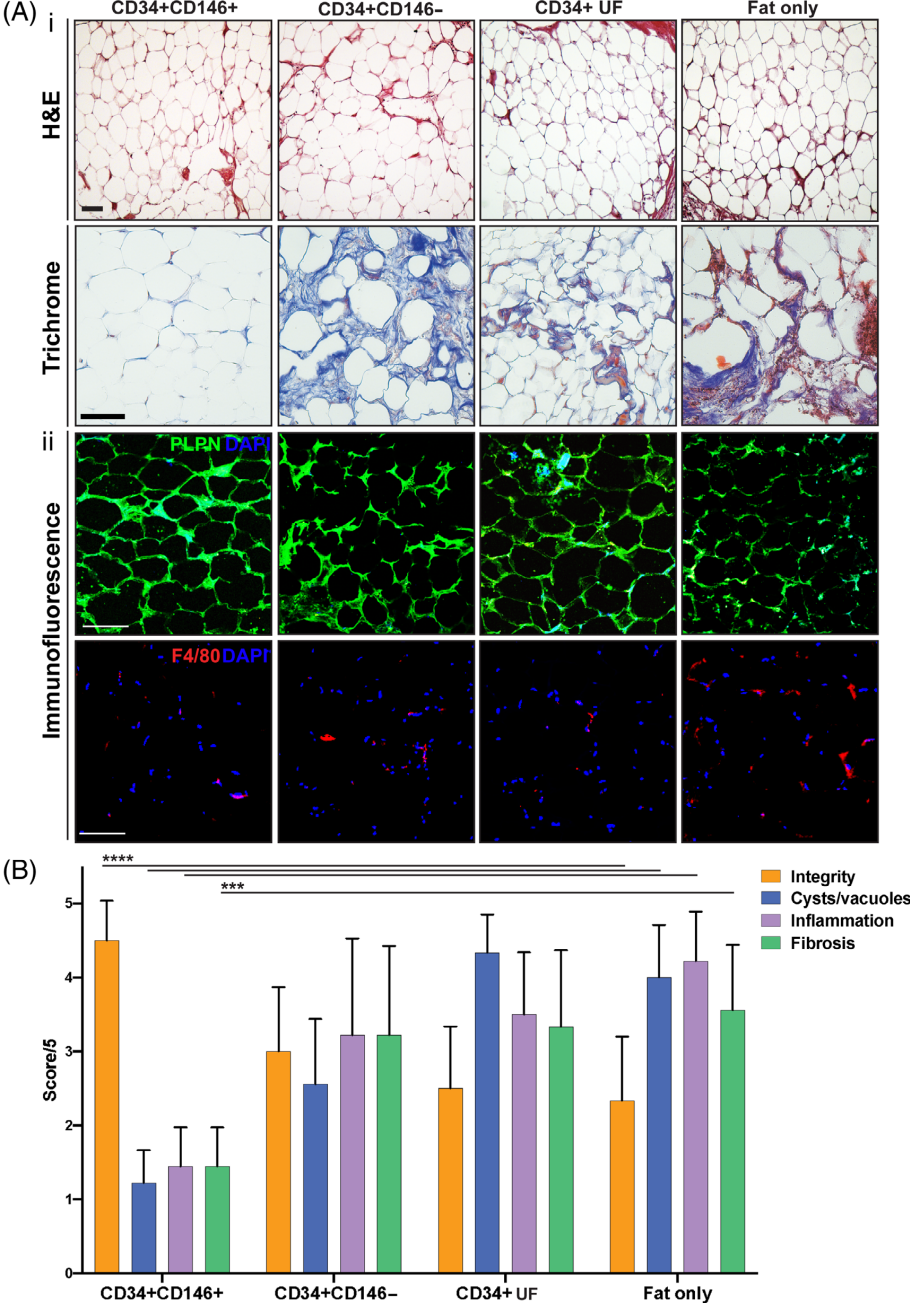
Histological evaluation of explanted fat grafts 8 weeks postgrafting. A, Representative images of fat grafts enriched with CD34+CD146+ (far left), CD34+CD146− (middle left), CD34+ UF (middle right) ASCs, and fat alone (“fat only,” far right) stained with H&E (A[i], top row), Masson's Trichrome (A[i], bottom row), perilipin (green) and DAPI (blue) (A[ii], top row), and F4/80 (red) (A[ii], bottom row). H&E and Trichrome staining indicated least fibrosis and greatest integrity in the CD34+CD146+ group. Perilipin staining indicates live adipocytes within grafted adipose tissue. Immunofluorescent images revealed perilipin stained adipocytes in all groups of mice. Fat enriched with CD34+CD146+ also had less staining for F4/80+, indicating fewer mouse macrophages within the grafted tissue. Scale bars = 100 μm. B, Blinded rating of H&E‐stained slides revealed that grafts enriched with CD34+CD146+ ASCs were of greater integrity, had fewer less cysts/vacuoles, less inflammation, and less fibrosis (***P < .001, ****P < .0001). ASC, adipose‐derived stromal cell; DAPI, 4′,6‐diamidino‐2‐phenylindole; UF, unfractionated

H&E‐stained sections of explanted grafts from the four groups of mice exhibited different histological characteristics 8 weeks postgrafting (Figure 5A[i], top row). The fat grafts enriched with CD34+CD146+ ASCs had greater integrity (****P < .0001), fewer cysts/vacuoles (****P < .0001), less inflammation (****P < .0001), and less fibrosis (***P < .001) than fat grafts enriched with CD34+CD146− ASCs, CD34+ UF ASCs, or fat alone (Figure 5B). Since dead adipocytes cannot be distinguished from living adipocytes using H&E, perilipin staining was used to label viable adipocytes. While grafted fat in all groups of mice contained viable adipocytes, more perilipin staining was observed in mice grafted with CD34+CD146+ and CD34+ UF ASCs (Figure 5A[ii], top row). Explants were also stained with F4/80+ to label macrophages. We noted grafts enriched with CD34+CD146+ had qualitatively less staining for F4/80, consistent with less inflammation (Figure 5A[ii], bottom row). Last, Masson's trichrome staining was used to further visualize fibrosis within the grafted fat. Also consistent with ratings of the H&E‐stained slides, fat grafts enriched with CD34+CD146+ ASCs had the least staining for collagen (blue) and thus the least fibrosis. The degree of fibrosis was comparable between grafts enriched with CD34+CD146− ASCs and fat grafted without ASC enrichment (Figure 5A[i], bottom row).

### 3.4 Improved vascularization of fat grafts enriched with CD146+ ASCs

**FIGURE 6:**
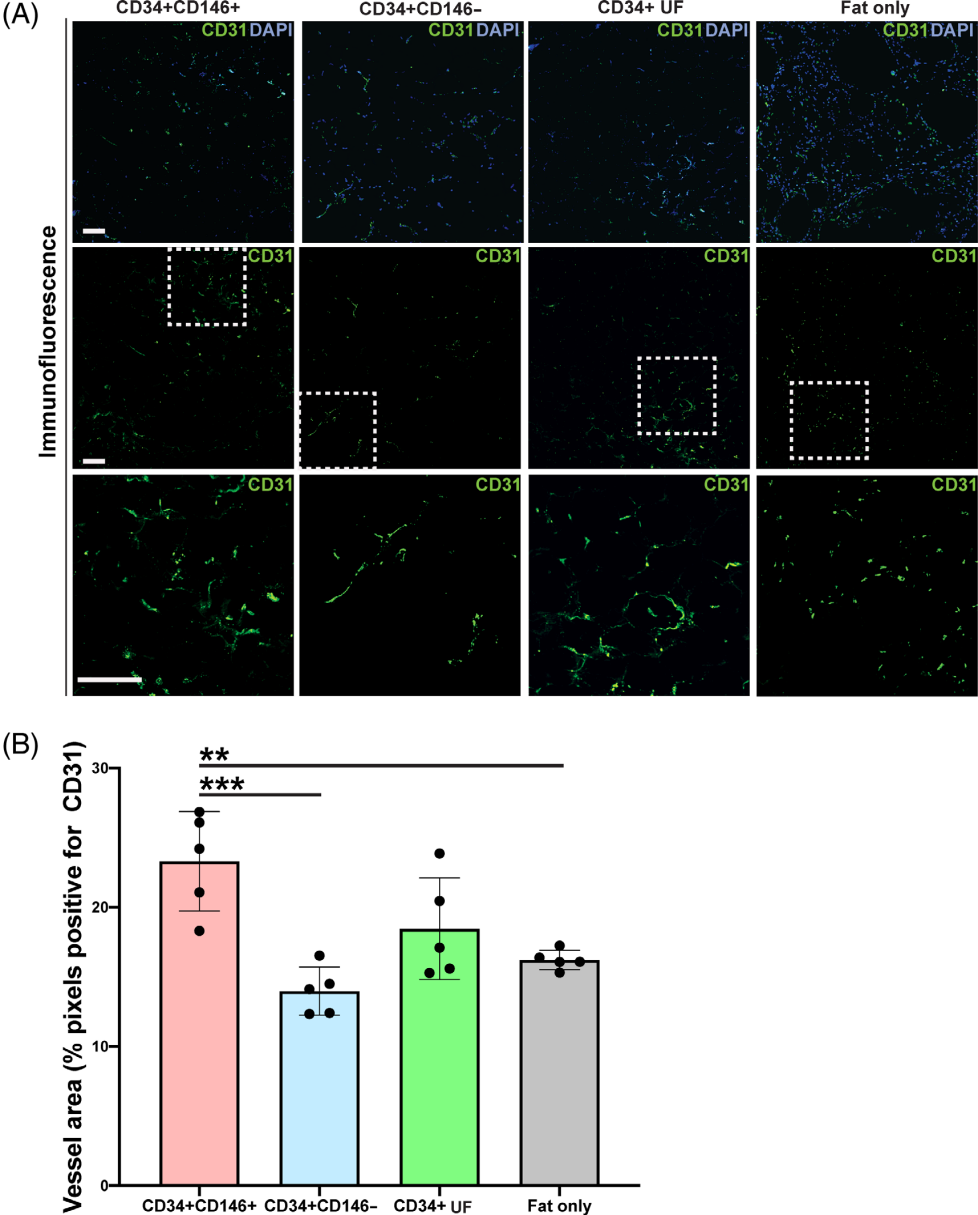
Vascularization of explanted fat grafts 8 weeks postgrafting. A, Immunofluorescence staining for endothelium using CD31 (green) in fat grafts enriched with CD34+CD146+ (far left), CD34+CD146− (middle left), CD34+ UF (middle right) ASCs, or not enriched alone (“fat only,” far right) shown at low magnification with DAPI (blue) (top row), with CD31 alone (middle row), and of the selected ROI (white dotted box) at high magnification (bottom row). Scale bars = 100 μm. B, Fat graft vascularization was quantified as ROI area occupied by CD31‐positive pixels. Fat enriched with CD34+CD146+ ASCs had greatest vascularization, indicated by increased CD31 immunofluorescence staining compared to fat enriched with CD34+CD146− or CD34+ UF ASCs, and grafts not enriched with ASCs and fat alone; n = 5 per group (*P < .05, **P < .01). ASC, adipose‐derived stromal cell; DAPI, 4′,6‐diamidino‐2‐phenylindole; ROI, region of interest; UF, unfractionated

To evaluate how enrichment of fat grafts with the three ASC subpopulations influenced graft vascularization, we performed CD31 immunohistochemical staining and assessed the areas that were CD31−pixel positive (Figure 6A, top, middle, and bottom rows). The results indicated that fat enriched with CD34+CD146+ ASCs was significantly more vascularized 8 weeks postgrafting than fat grafts enriched with CD34+CD146− ASCs (***P < .001) and fat alone (**P < .01), and trended toward being more vascularized that fat enriched with CD34+ UF ASCs (Figure 6B).

## 4 DISCUSSION

ASCs are a collection of distinct stem and progenitor subpopulations that have unique gene and protein expression profiles and functional capacities,^9^, ^31^ and defining ASC heterogeneity is a topic of current investigation. Given the poor retention of grafted fat, identification of ASC subpopulations with enhanced proangiogenic qualities may improve the efficiency and regenerative potential in current fat grafting procedures. Here, we identified CD146 as a marker of ASCs with proangiogenic qualities in vitro and in vivo; CD34+CD146+ ASCs have increased expression of VEGF, ANGPT1, and FGF, an enhanced capacity to induce endothelial tube formation, and the ability to improve the retention and vascularization of grafted fat.

Cluster differentiation 146 (CD146), also known as Melanoma Cell Adhesion Molecule (Mel‐CAM, MCAM), is a membrane glycoprotein that functions as a receptor for lamin alpha 4 to mediate cell adhesion. CD146 is expressed within a number of cell types, including vascular endothelial cells, smooth muscle cells, and pericytes.^32^ The exact biological role of CD146, however, is poorly defined. CD146+ cells play a role in implantation, placentation, and tumor progression‐possibly through enhancing the interaction between endothelial and melanoma cells.^33^ Umbilical cord MSCs positive for CD146 have enhanced tube‐forming abilities and coexpress the Notch ligand Jagged1 (JAG1), a known potent regulator of blood vessel maturation.^11^ Pericytes, cells believed to give rise to MSCs, express CD146, and transplantation of purified pericyte populations promotes tissue repair and angiogenesis.^30^ Like pericytes, ASCs are predominantly associated with vascular structures within adipose tissue, but unlike pericytes, they are CD34+.^34^ Recent work has suggested that CD146 may also mark a proangiogenic subset of ASCs (CD34+) in human adipose tissue.^35^, ^36^ The angiogenic qualities of this subpopulation, however, have only been demonstrated using in vitro assays.^35^, ^36^ Our work is the first to demonstrate the angiogenic effects of the CD34+CD146+ subpopulation in vivo in the setting of fat grafts.

The mechanisms by which ASCs promote fat graft vascularization are thought to be largely mediated via paracrine signaling.^4^ Here, we show that CD34+CD146+ ASCs highly express VEGF, a potent proangiogenic factor.^37^ VEGF induces endothelial mitosis by acting though mitogen‐activated protein kinases,^38^ and also promotes endothelial cell migration, together leading to angiogenesis of existing vessels.^39^ The CD34+CD146+ ASC subpopulation also highly express ANGPT1, another endothelial cell‐specific growth factor. ANGPT is reported to stabilize blood vessels and counteract VEGF‐induced blood vessel leakage.^40^ Thus, together, VEGF and ANGPT1 have an additive effect on angiogenesis.^40^ Interestingly, the CD34+CD146+ ASC subpopulation expresses more ANGPT1 than CD34−CD146+ pericytes, and may therefore be of greater therapeutic utility. Finally, the surface marker CD146 can be cleaved by metalloproteases and released by CD146+ cells as a soluble molecule.^41^, ^42^ In this form, CD146 can act chemotactically to enhance formation of vascular structures by endothelial cells and promote neovascularization in a hind limb ischemia model in rats.^43^ The enhanced vascularization observed when grafted fat is enriched with CD34+CD146+ ASCs likely also explains the observed reduced fibrosis and inflammation when fat grafts are enriched with this ASC subpopulation. Fat necrosis is an inflammatory process which in time leads to tissue fibrosis. Improved delivery of oxygen and nutrients by effective vascularization in the early stages postgrafting may promote adipocyte survival, reduce inflammation, and prevent tissue fibrosis.

Fat grafting procedures are commonly performed surgeries; in the United States, it is estimated that more than 400 000 liposuction procedures are performed each year.^44^ Fat grafting is being adopted not only as a technique for restoring contour deformities, but also as a method by which to mitigate fibrosis and the effects of radiation damage on skin. Despite this growing popularity, fat retention rates are variable, especially in irradiated tissue where pathological fibrosis severely diminishes the dermal vascular supply. Enriching fat grafts with proangiogenic ASCs may confer enhanced retention and address the unpredictability of fat grafting. The CD146+ ASC subpopulation, which represents a significant proportion of CD34+ ASCs, may be of particular benefit in this setting. Beyond fat grafting, this proangiogenic ASC subpopulation may help promote vascularization and minimize fibrosis in wounded or irradiated skin. As such, our future aims include investigating the role of CD146+ ASCs in these settings. Finally, while CD146 may mark an angiogenic subset of ASCs, it is likely that other ASC populations play comparable or complementary functions in angiogenesis. We have previously identified a separate population of proangiogenic ASCs, distinguished by the surface marker CD248.^45^ Although not investigated in this article, future work may consider the degree to which ASCs coexpress both CD146 and CD248 and how their roles overlap or interact to promote fat graft retention.

## 5 CONCLUSION

We have identified a subpopulation of ASCs that express the CD146 surface marker and exhibit enhanced regenerative qualities. This group of CD34+CD146+ ASCs express higher levels of proangiogenic growth factors, promote endothelial tube formation, and enhance fat graft retention. Enrichment of lipoaspirate with CD34+CD146+ ASCs may thus help to improve the efficiency and outcomes of current fat grafting procedures.

## CONFLICT OF INTEREST

A.M. declared Consultancy for Allergan, AxoGen, Sientra, and Stryker. The other authors declared no potential conflicts of interest.

## AUTHOR CONTRIBUTIONS

M.R.B.: conception, design, preparation, analysis and interpretation of the data, revision of the manuscript; C.B., S.V., D.C.W.: conception, design, preparation, revision of the manuscript; R.A.P., J.S., N.M.D.D., S.A., A.H.S., A.M., D.N.: assistance in data collection and assembly; M.T.L.: preparation guidance, revision of the manuscript; All authors reviewed and approved of the final manuscript.

## Supporting information


**Fig. S1** Fluorophore minus one controls. FACS plots showing “fluorophore minus one” (FMO) controls for; all three lineage antibodies in a single FMO (top grey box), CD146‐PECy7 (middle grey box), and for CD34‐AF488 (bottom grey box). Gating strategy used for in vitro and in vivo experiments was based on these FMOs.

**Fig. S2** (A) Gating strategy used to isolate CD34‐CD146+ cells. CD34‐CD146+ subpopulation was 41.4% of lineage negative live single cells that were CD34‐. (B) Gene Expression of ASCs and pericytes. Expression of three potent pro‐angiogenic genes (VEFG ‐ vascular endothelial growth factor, ANGPT1 ‐ angiopoietin‐1, FGF ‐ fibroblast growth factor‐2) between CD34 + CD146+, CD34+ UF ASCs (classic adipose MSCs), and CD34‐CD146+ pericytes. CD34 + CD146+ ASCs express equivalent levels of VEGF and FGF, but significantly more ANGTP1 than CD34‐CD146+ pericytes (*P < 0.05). CD34 + CD146+ ASCs express significantly more VEGF than CD34+ UF ASCs (*P < 0.05), and CD34‐CD146+ pericytes express significantly more FGF than CD34+ UF ASCs (*P < 0.05).

## Data Availability

The data that support the findings of this study are available on request from the corresponding author.
