# Supplementary figures and images for: CD34+CD146+ adipose‐derived stromal cells enhance engraftment of transplanted fat
Source: Stem Cells Transl Med. 2020 Jun 15;9(11):1389–400. doi: 10.1002/sctm.19-0195 (PMC7581443; doi:10.1002/sctm.19-0195)

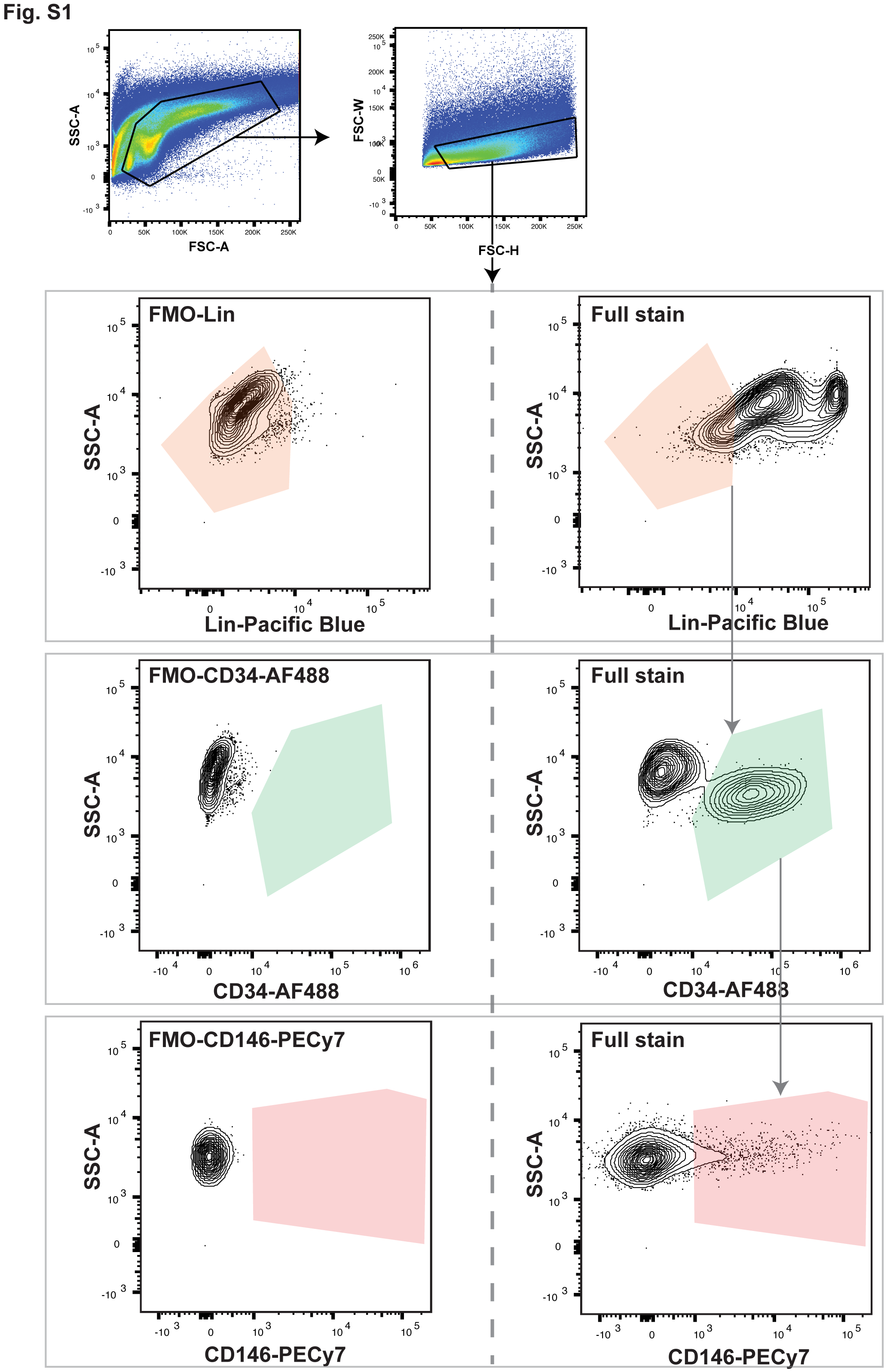

Supplement: Supplementary file 1 — Fig. S1 Fluorophore minus one controls. FACS plots showing “fluorophore minus one” (FMO) controls for; all three lineage antibodies in a single FMO (top grey box), CD146‐PECy7 (middle grey box), and for CD34‐AF488 (bottom grey box). Gating strategy used for in vitro and in vivo experiments was based on these FMOs. [file SCT3-9-1389-s001.tif]

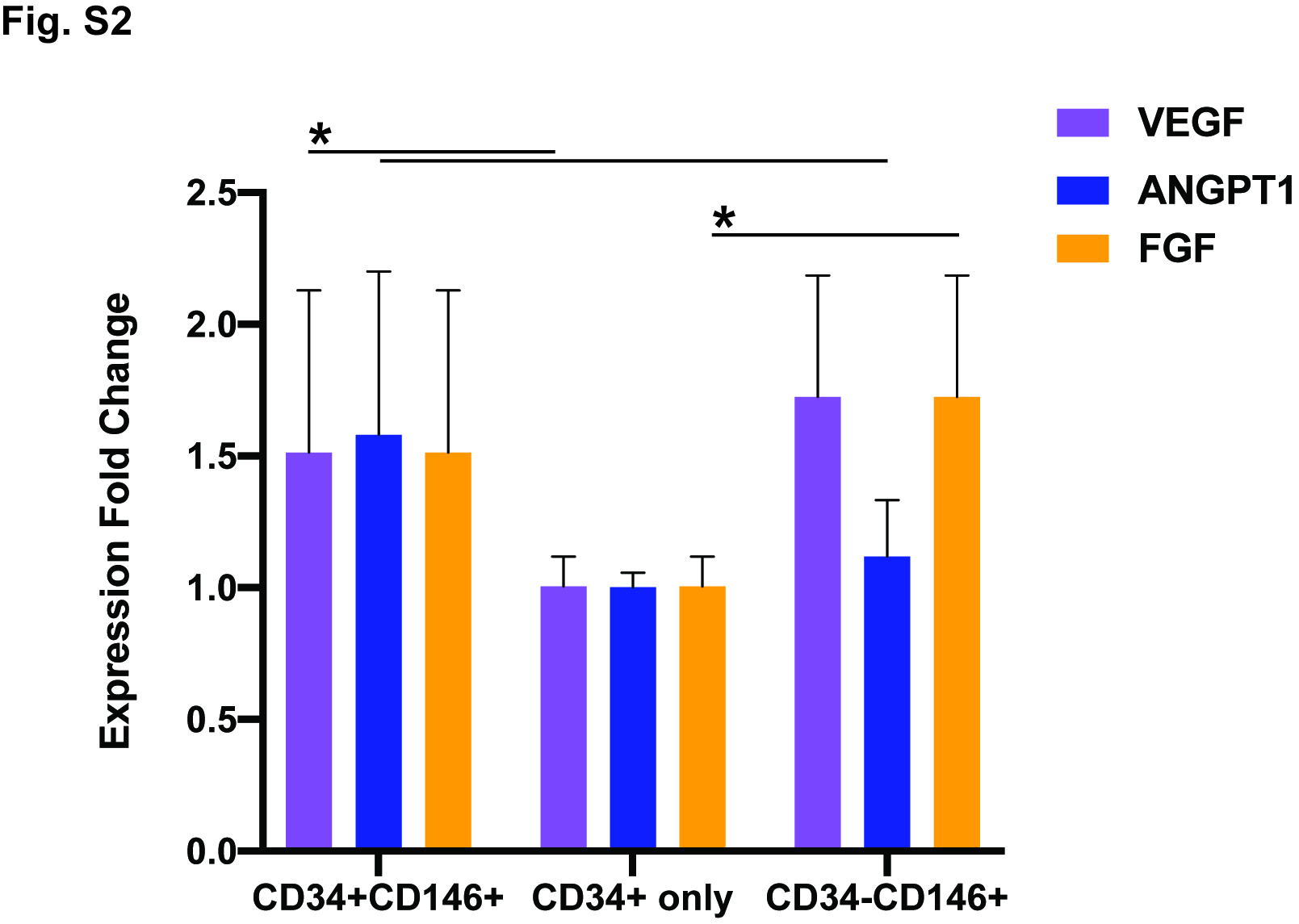

Supplement: Supplementary file 2 — Fig. S2 (A) Gating strategy used to isolate CD34‐CD146+ cells. CD34‐CD146+ subpopulation was 41.4% of lineage negative live single cells that were CD34‐. (B) Gene Expression of ASCs and pericytes. Expression of three potent pro‐angiogenic genes (VEFG ‐ vascular endothelial growth factor, ANGPT1 ‐ angiopoietin‐1, FGF ‐ fibroblast growth factor‐2) between CD34 + CD146+, CD34+ UF ASCs (classic adipose MSCs), and CD34‐CD146+ pericytes. CD34 + CD146+ ASCs express equivalent levels of VEGF and FGF, but significantly more ANGTP1 than CD34‐CD146+ pericytes (*P < 0.05). CD34 + CD146+ ASCs express significantly more VEGF than CD34+ UF ASCs (*P < 0.05), and CD34‐CD146+ pericytes express significantly more FGF than CD34+ UF ASCs (*P < 0.05). [file SCT3-9-1389-s002.tif]
